# Cytotoxic and Bactericidal Effect of Silver Nanoparticles Obtained by Green Synthesis Method Using *Annona muricata* Aqueous Extract and Functionalized with 5-Fluorouracil

**DOI:** 10.1155/2018/6506381

**Published:** 2018-10-15

**Authors:** María del Carmen Sánchez-Navarro, Claudio Adrian Ruiz-Torres, Nereyda Niño-Martínez, Roberto Sánchez-Sánchez, Gabriel Alejandro Martínez-Castañón, I. DeAlba-Montero, Facundo Ruiz

**Affiliations:** ^1^Facultad de Estomatología, Universidad Autónoma de San Luis Potosí (UASLP), Avenida Manuel Nava 2, Zona Universitaria, 78290 San Luis Potosí, Mexico; ^2^Facultad de Ciencias, Universidad Autónoma de San Luis Potosí (UASLP), Avenida Manuel Nava 6, Zona Universitaria, 78290 San Luis Potosí, Mexico; ^3^Instituto Nacional de Rehabilitación LGII, CENIAQ, Calzada México Xochimilco No. 289, Colonia Arenal de Guadalupe, Delegación Tlalpan, 14389 Ciudad de México, Mexico

## Abstract

Nanomaterials obtained by green synthesis technologies have been widely studied in recent years owing to constitute cost-effective and environmental-friendly methods. In addition, there are several works that report the simultaneous performance of the reducer agent as a functionalizing agent, modifying the properties of the nanomaterial. As a simple and economical synthesis methodology, this work presents a method to synthesize silver nanoparticles (AgNPs) using *Annona muricata* aqueous extract and functionalized with 5-fluorouracil (5-FU). The processes of reduction, nucleation, and functionalization of the nanoparticles were analyzed by UV-Vis absorption spectroscopy, and it was found that they are the function of the contact time of the metal ions with the extract. The structural characterization was carried out by transmission electron microscopy (TEM) and X-ray diffraction patterns (XRD). The antibacterial properties of the synthetized nanomaterials were tested using minimum inhibitory concentration (MIC) and minimum bactericidal concentration (MBC) against *Enterococcus faecalis*, *Staphylococcus aureus*, and *Escherichia coli* growth.

## 1. Introduction

The use of nanoparticles as nanodelivery vehicles has received quite great interest in the medical sector in recent years owing to the fact that, by biochemical engineering, it is possible to design multifunctional nanostructured biomaterials to deliver specific drugs to target tumor or cancer cells [1]. The best performance of nanobiomaterials with respect to other types of biomaterials is due to quite high compatibility and adaptability to biological systems, which additionally represents nonviral systems, constituting promising tools in biomedicine research. A clear example of this fact is silver nanoparticles; due to the application of these types of materials to biological systems, there has been development of numerous nanodelivery vehicles, in view of their intrinsic properties, biocompatibility, and antimicrobial capacity [2]. It is necessary to improve material properties and biocompatibility for a more efficient yield in drug delivery to a specific target, avoiding a wide distribution of the medicine. In light of this, the material functionalization and organometallic science by the development of covalent nets or polymeric functionalization have modified the interaction of inorganic surface on metallic NPs with the surrounding media, improving their performance in biomedicine [3, 4].

5-Fluorouracil (5-FU) is a potent broadly used antimetabolite for cancer treatments such as advanced oral cancer [5]. Nevertheless, several limitations exist on its use related to the short half-life, lack of control on selective delivery, and ample diffusion on body, limiting its antitumor applicability. Therefore, through the functionalization of nanostructured materials with 5-FU, it has remarkably improved the utility of the drug and produced novel and proficient tools for oncologic research [6].

Currently, ample spectrum of different methodologies for silver nanobiomaterials obtained and functionalization for biomedical purposes exists [7]. However, in the previous years, a remarkable interest in the green synthesis methods of nanobiomaterials has been rising in response which represents environmental-friendly methods, low-toxic methodologies, cost-effective alternatives, and one-step NP synthesis-functionalization method [8]. In general, nanomaterial synthesis by “green methods” considers three main characteristics according to Raveendran et al. [9] as follows: (1) solvent friendly as a reaction medium; (2) environmentally beneficial reducing agents; and (3) use of nontoxic material as capping agents [9]. In addition, different reports had reported not only the achievement of obtaining nanoparticles by green synthesis but also discussed the medicinal properties of the materials associated with the active ingredients present in the natural extracts used, which has promoted a scientific focus on the biological activities of these kinds of substances [10, 11]. Furthermore, in relation to the information described above, the synthesis of silver nanomaterials through an efficient, economically cheap, and environmentally safe method has become an important research area in nanobiotechnology. Therefore, currently, diverse plant extracts have been used as excellent bioreducing agents in the synthesis of silver nanoparticles (AgNPs) [12, 13].

The extract *Annona muricata*, generally known as guanabana, is largely distributed in tropical areas of South America and North America; all fractions of the *A. muricata* tree are widely used as traditional medicines against human diseases, including cancer and infections. The antiinflammatory, hypoglycemic, sedative, smooth muscle relaxant, hypotensive, and antispasmodic effects are attributed to the leaves, barks, and roots of *Annona muricata*. The leaves of this plant are also employed against tumors and cancer in South America and tropical Africa [14]. Phytochemical evaluations of the *Annona muricata* plant have shown the presence of alkaloids, megastigmanes, flavonol triglycosides, phenolics, cyclopeptides, essential oils, and some minerals such as K, Ca, Na, Cu, Fe, and Mg [14].

In the present work, the synthesis of silver nanoparticles using *Annona muricata* aqueous extracts as a bioreducing agent is reported. The materials were characterized in terms of their optical properties, crystallinity, morphology, hydrodynamic radius, and surface charge. The antibacterial capacities of the materials were evaluated by their bactericidal effect against oral microorganisms such as *E. Faecalis*, *S. Mutans*, *S. Oralis*, *S. Aureus*, and *E. Coli* by minimum inhibitory concentration (MIC) and minimum bactericidal concentration (MBC). Moreover, the evaluation of the cytotoxicity of the samples was done by the cellular viability of fibroblast cells using MTT assay and fluorescent microscopy.

## 2. Materials and Methods

### 2.1. Reagents

*A. muricata* leaves were purchased from a local supermarket; silver nitrate (AgNO_3_) and 5-FU were obtained from Sigma-Aldrich; serological pipettes (5, 10, and 25 mL) and 50 mL centrifuge tubes were purchased from Santa Cruz Biotechnology, Inc.; 25 cm^3^ cell-culture flask were purchased from Corning®; and 48-well cell-culture cluster were purchased from Costar®.”

Dulbecco's Modified Eagle Medium (DMEM), phosphate-buffered saline (PBS) of pH 7.4, fetal bovine serum (FBS), penicillin/streptomycin, and doxorubicin were purchased from Gibco®. The following bacteria were used: *Enterococcus faecalis* (ATCC 29212), *Staphylococcus aureus* (ATCC 29213), and *Escherichia coli* (ATCC 25922). Human fibroblasts were donated by Dr. Roberto Sánchez-Sánchez, biotechnology laboratory of Instituto Nacional de Rehabilitación LGII, CENIAQ (Ciudad de México).

### 2.2. AgNP Synthesis

The synthesis of the materials was carried out by the use of *Annona muricata* extract as a bioreducing agent based on the previously reported method by Santhosh et al. [15]. Initially, the leaves were washed with deionized water to remove impurities. After being crushed in a blender, 5 g of the powder was previously ground in 125 mL of deionized water and was boiled to the boiling point. After obtaining the infusion of the extract, in a separate vessel, Ag salt was added and the process of reduction and formation of the nanoparticles began, which was evidenced by the immediate color change, indicating the formation of the same ones. The color of the mixture changed from pale brown to dark brown for AgNPs, and no synthetic reagents were required for this synthesis. Finally, the NP sedimentation was induced by centrifugation and washed with ethanol three times.

### 2.3. Silver NP Functionalization with 5-Fluorouracil

Prior to Ag nanoparticle synthesis and washing, 50 ml of NP solution was taken in a vessel; subsequently, 0.5 g of 5-fluorouracil was added, and the solution was sonicated for 20 min.

### 2.4. Physical Characterization Methods

UV-Vis absorption spectra were obtained using the S2000 UV-Vis spectrometer (OceanOptics, Inc.). Transmission electron microscopy (TEM) images were obtained at 100 kV using a JEOL-1230. The hydrodynamic radius and Z-potential of the samples were measured with a Nanosizer DLS. Furthermore, the X-ray diffraction (XRD) patterns were obtained using a GBC-Difftech MMA diffractometer with filtered CuK*α* (*λ* = 1.54 Å) radiation.

### 2.5. Antibacterial Activity of AgNPs

The antimicrobial activities of the AgNPs were confirmed via minimum inhibitory concentration (MIC) and minimum bactericidal concentration (MBC) against strains of *E. faecalis* (ATCC 29212), *S. aureus* (ATCC 29213), and *E. coli* (ATCC 25922); they were studied at 0.5 of the McFarland Scale determined with a colorimeter (LaMotte Smart3) according to the standard microdilution method (CLSI M100-S25, January 2015) [16].

### 2.6. Cytotoxicity Assay

With respect to cytotoxicity assays, human fibroblasts isolated from the dermis were cultured and stored in liquid nitrogen at −196°C, in order to retain the viability of the cells. A cell culture was carried out in 25 cm^3^ plates (Costar®), until the surface of the culture vessel was covered, waiting for the formation of a monolayer (layer thickness of one cell). Previously, subcultures were carried out for three weeks using the DMEM culture medium (Gibco®), which contains specific proteins essential for cell survival, development, and proliferation. Subsequently, the incubation was performed at 37°C under a CO_2_ atmosphere using a NUAIRE Autoflow Ir Water-Jacketed CO_2_ incubator; medium changes were made and observed on an inverted microscope (Axio-Zeiss). Once the desired cell confluence was obtained, the cultures were treated with trypsin (a proteolytic enzyme that degrades the extracellular matrix and sequesters the calcium ion, which is essential for cell adhesion). In addition, by gentle agitation after 5–7 min, the cells detached, and the cell suspension required to calculate the number of cells present in a certain volume were obtained and analyzed in a Neubauer chamber. Furthermore, 20,000 cells per cm^2^ were seeded in 48-well plates and cultured for 24 hrs. With the purpose of material cytotoxicity assays performed with calcein and ethidium homodimer (Thermo®), different dilutions of silver nanoparticles synthesized by a green method were made using an extract of *Annona muricata*. The treatment groups used to perform the cytotoxicity test are shown in Table 1:

The viability of human fibroblasts after exposure to AgNPs was evaluated by the amount of viable cells stained by MTT assay. The human fibroblasts were plated in 96-well plates and exposed to AgNPs, AgNPs + 5-FU, 5-FU, and *Annona muricata*. Cells were added into the medium at concentrations of 30 *μ*g/ml (ppm) maintained in a humidified atmosphere at 37°C and 5% CO_2_. After 24 and 48 h, the medium was removed from each well, replaced with a new medium with MTT solution in an amount of 10% of culture volume, and incubated for 4 h at 37°C until a blue-colored formazan product developed. The resulting formazan product was dissolved in DMSO, and the absorbance was measured at 570–690 nm by using a Synergy HTX Multi-Mode Microplate reader (BioTek Instrument, Inc.).

## 3. Results and Discussion

### 3.1. Structural Characterization of AgNPs

#### 3.1.1. Ultraviolet-Visible (UV-Vis) Absorption Spectroscopy

**Figure 1:**
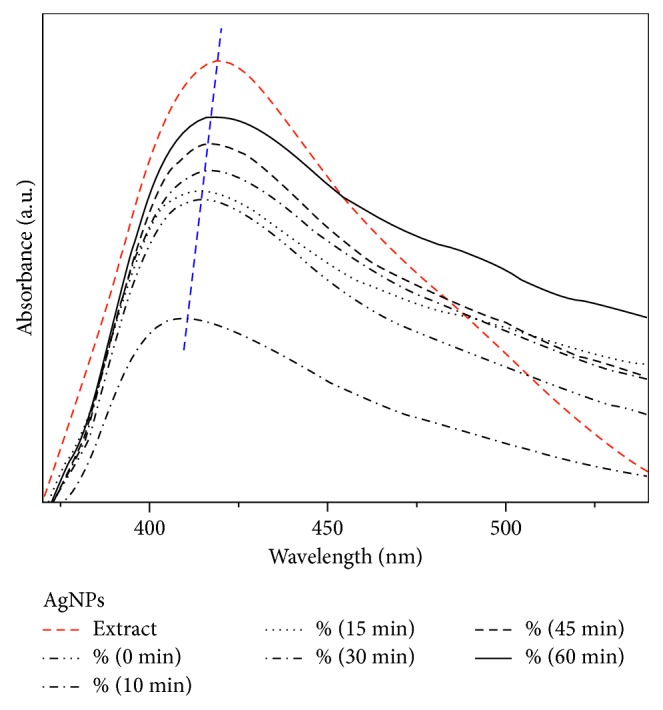
UV-Vis absorption spectra of silver nanoparticles at different time periods and their reaction with *A. muricata* extract at 1000 *μ*L.

The UV-Vis spectra of the materials were made in order to follow the nucleation process of the particles as a function of the contact time with the extract. In Figure 1, a band at 425 nm can be observed, which is associated with the surface plasmon of silver nanoparticles. The intensity of the band increases as the contact time of the metal ions with the extract is increased, having its maximum intensity at 60 min. Additionally, it is possible to observe a thinning of the band of the final solution with respect to the initial solution. This thinning can be associated to two phenomena; firstly, the influence of the symmetry of the particles and their size distribution on their optical properties has been previously reported, where irregular particles (nonspherical) will show two or more surface plasmon bands, which will result in the widening of the band; this is due to the fact that as the reduction process begins, the nucleation and the generation of the primary particles will take place, which will have irregular shapes and smaller diameters, explaining the blue shift of the band in the initial solutions and a red shift in the final solutions where the process of nucleation and generation of the particles is concluded [17, 18].

On the other hand, due to the fact that the nanoparticle formation process is interrupted, there will possibly be several particle sizes influencing the excitation surface plasmon peak. Finally, another phenomenon involved to this fact is the band of absorption of the extract, which is at 435 nm, affecting the band position and possibly influencing the red shift. In relation to this, the preservation of the organic agent on the surface of silver nanoparticles evidenced its role as a functionalizing agent.

#### 3.1.2. Morphological Characterization: TEM

**Figure 2:**
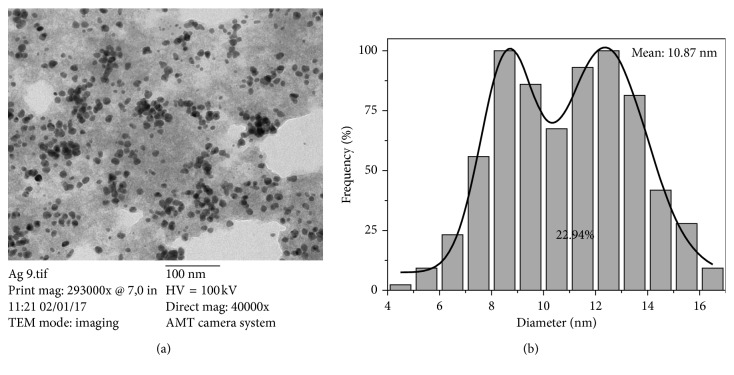
(a) TEM photomicrography and (b) differential size distribution of silver nanoparticles biosynthesized using *A. muricata* extract.

Figure 2(a) presents the TEM images and histograms of the synthetized materials. AgNPs present a quasi-spherical morphology arranged in isolated clusters. The differential distribution corresponding to this sample displays a particle size range between 4.54 and 16.48 nm with an asymmetrical geometry and a bimodal distribution (Figure 2(b)). The average particle size and coefficient of variation (CV%) calculated for this sample were 10.87 nm and 22.94%, respectively. The statistical parameters obtained are shown in Table 2.

#### 3.1.3. Dynamic Light Scattering (DLS) and Zeta Potential

Table 3 displays the values of particle size and Z-potential obtained for the particles. The acquired value for particle size was 16.46 ± 0.46, slightly differing from the obtained diameter mean of 10.87 from TEM images analysis, indicating the association of the value corresponding to DLS analysis to the hydrodynamic radius of the particles and corroborating the results shown in size statistical analysis. The measured Z-potential of the sample displays a value of −27.3 ± 1.22 mV, which represent an electrostatic repulsion between the particles as shown in the TEM image corresponding to this sample, where most of the NPs are well dispersed (Table 3).

#### 3.1.4. X-Ray Diffraction

**Figure 3:**
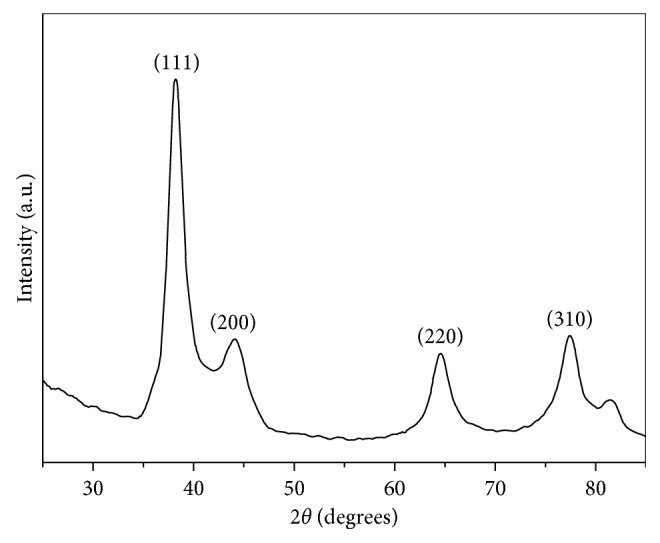
X-ray diffraction patterns of the Ag sample.

The X-ray diffractogram corresponding to AgNPs presents the peaks associated with the Ag cubic phase at 38.23, 44.18, 64.74, 75.51, and 81.74°, which can be indexed as the (111), (200), (220), (311), and (222) planes (JCPDS File No.: 04–0783), respectively, illustrating with this information the presence of crystalline silver nanoparticles (Figure 3).

### 3.2. Antimicrobial Activity of AgNPs

In this study, MIC values were obtained for the AgNPs tested against *E. coli* (ATCC 25922), *S. aureus* (ATCC 29213), and *E. faecalis* (ATCC 292129). The results are presented in Table 4, where the MIC of the AgNPs synthesized by *Annona muricata* presents a lower antibacterial activity in comparison with 5-fluoracil, but the functionalization/combination of the silver nanoparticles with 5-fluoracil showed a synergism because all the strains were inhibited at a concentration of 1.95 Dg/ml or less. With respect to the tree strains tested with AgNPs and 5-fluoracil, *Enterococcus faecalis,* which is a facultative anaerobic Gram-positive coccus, shows the lowest sensitivity in comparison with *E. coli* and *S. aureus* [19]; A. Manten and J. I. Terra were the first to report the antibacterial activity of antineoplastic drugs as well as the combination effects between the antibacterial and antineoplastic agents [19]. The results corresponding to 5-fluoracil alone displayed similar results compared to the data reported by Gieringer et al. [20], who found that concentrations of 0.8 *μ*g/ml or less inhibit all strains of *Staphylococcus aureus* [20].

### 3.3. Cytotoxicity Assay

#### 3.3.1. MTT Assay

The cell viability was evaluated by the cytotoxicity test and fluorescent microscopy in order to compare the effects produced on oral fibroblasts by green AgNPs and the presence and absence of 5-FU as the functionalization agent on the nanomaterials.

**Figure 4:**
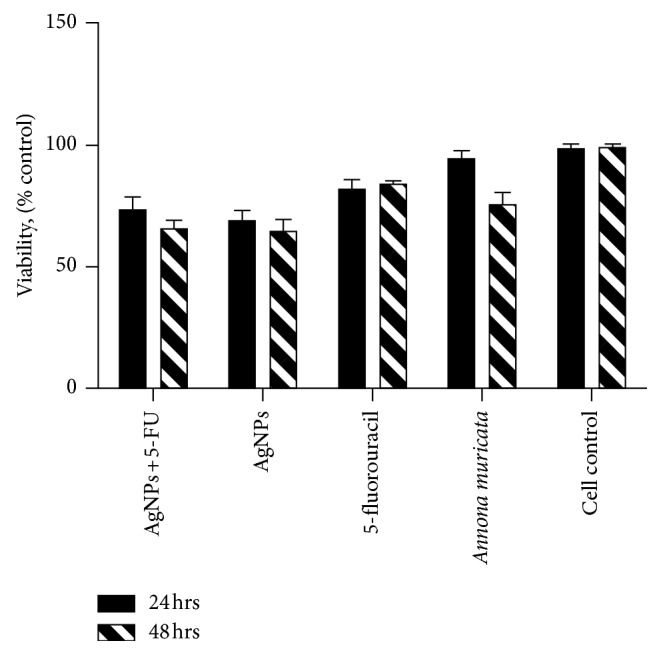
Graph of cytotoxicity of fibroblasts exposed to different concentrations of Ag nanoparticles for 24 and 48 hours.

The results corresponding to the cytotoxicity test show slight differences in cell viability between 24 and 48 hrs of cell exposition to AgNPs. It is possible to observe toxicity for silver nanoparticles at the highest concentrations used (Figure 4). AshaRani et al. [21] report similar results for lung fibroblasts in response to silver NPs exposition, arguing a cytotoxicity dependence on NPs concentration as well as cell-cycle detection [21]. Additionally, Ahmad et al. [22] evaluated the effect of silver and gold nanoparticles obtained by the green synthesis method in murine macrophages using concentrations in the range of 10–1000 *μ*g/mL, obtaining less cytotoxicity at a concentration lower than 80 *μ*g/mL [22]. Furthermore, the information presented illustrates a cytotoxicity-effect reduction of the nanomaterials by 5-FU functionalization, evidencing the biocompatibility improvement of the nanobiomaterials.

#### 3.3.2. Fluorescence Microscopy

**Figure 5:**
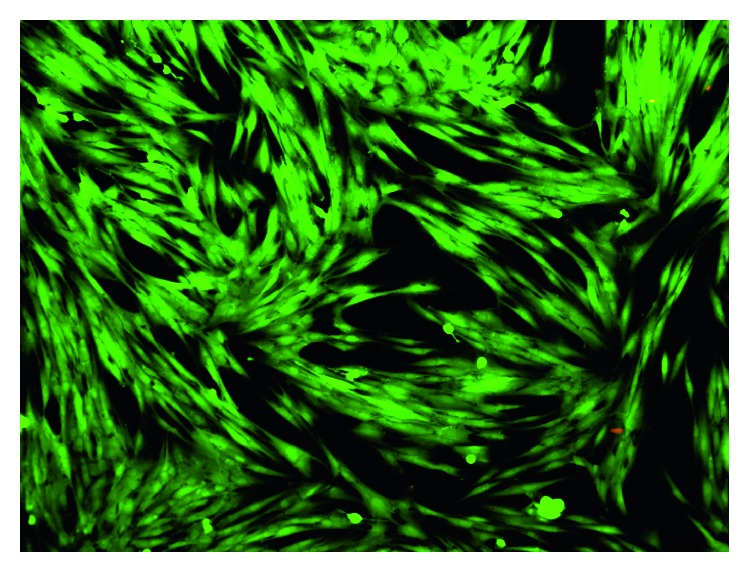
Fluorescent microscopy image of the control group of fibroblasts at 24 hrs.

**Figure 6:**
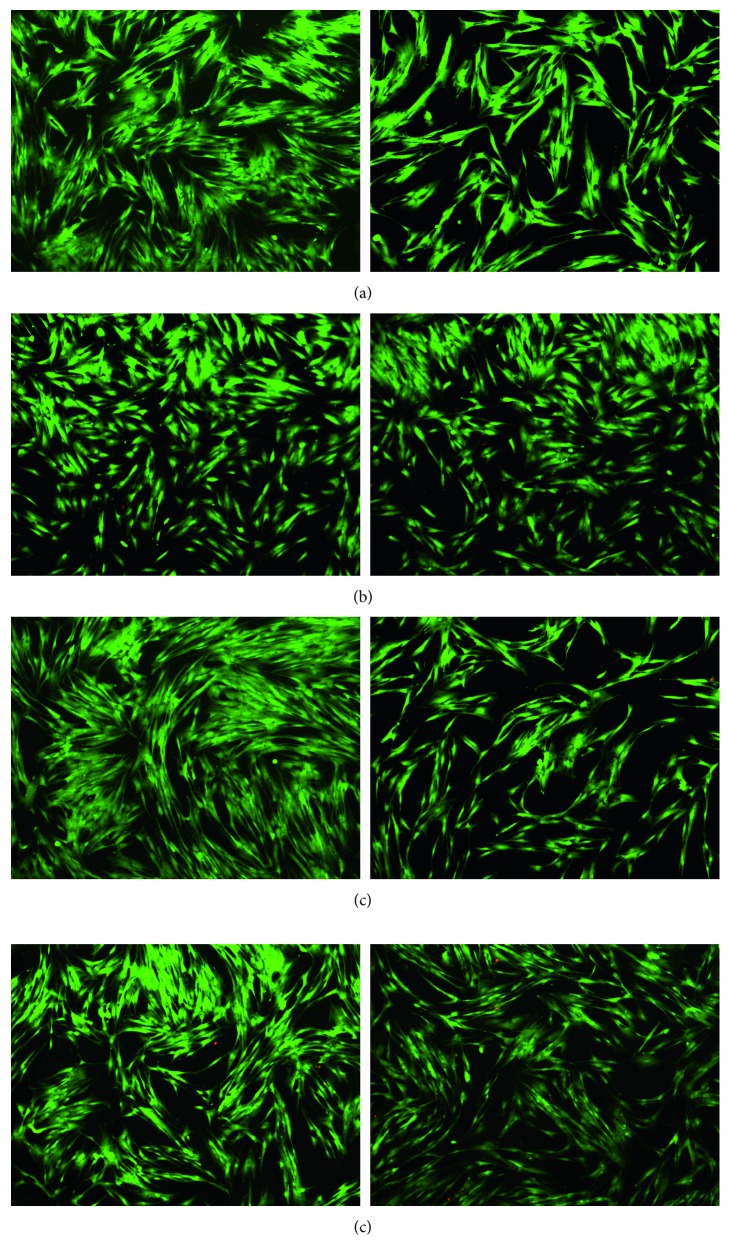
Fluorescent microscopy images of cytotoxicity assays in fibroblasts at 24 hrs and with respective concentrations at which it was evaluated (lowest and highest). (a) *A. muricata.* (b) 5-FU group. (c) AgNPs group. (d) Group of AgNPs and 5-FU.

Corresponding to the fluorescence microscopy analysis, it was observed that, at a lower concentration of treatments, there was more confluence and cell density compared to the control group (Figures 5 and 6). On the other hand, as concentrations increased, alterations in cells morphology and few cell extensions were observed. Moreover, an important characteristic observed at 24 is the presence of few dead cells for AgNPs exposition, indicating the role of the bioextract as a possible cytoprotective agent since it encapsulates the nanoparticles and 5-FU, preventing direct contact with cells, and additionally, the cell cultures remained viable with only the presence of the extract (Figures 6(b)–6(d)). Similarly, AshaRani et al. [21] previously reported different morphological changes, indicating unhealthy cells, few cell extensions, and restricted propagation patterns with respect to control [21].

## 4. Conclusions

In this work, it was demonstrated that, in the green synthesis of silver nanoparticles using *Annona muricata* as a bioreducer, the obtained NPs were characterized in terms of their optical properties, crystallinity, morphology, hydrodynamic radius, and surface charge. The UV-Vis monitoring of the AgNPs formation displayed the nucleation process of the particles and the increasing intensity of the silver characteristic band at 435 nm depending on time reaction. The nanoparticles presented a quasi-spherical shape with an average particle size of 10.87 nm and a hydrodynamic radius of 16.46 ± 0.46 nm. The Z-potential obtained had a value of −27.3 ± 1.22 mV, demonstrating repulsion between the particles and good colloidal stability of the material. The antimicrobial properties of the materials showed a great inhibition against Gram-positive and Gram-negative bacteria. The cytotoxicity of the NPs at 24 and 48 hrs displayed an increment in cell viability associated with the particles functionalization by 5-FU, and only a few dead cells at 24 hrs were observed in the fluorescence microscopy images.

## Figures and Tables

**Table 1:** Treatments and different concentrations used in the cytotoxicity tests carried out in fibroblasts.

Concentrations	C1	C2	C3	C4
*Annona muricata*	100 *μ*g/mL	75 *μ*g/mL	50 *μ*g/mL	25 *μ*g/mL
5-FU	20 *μ*g/mL	15 *μ*g/mL	10 *μ*g/mL	5 *μ*g/mL
AgNPs	32 *μ*g/mL	24 *μ*g/mL	16 *μ*g/mL	8 *μ*g/mL
AgNPs + 5-FU	23 *μ*g/mL	18 *μ*g/mL	13 *μ*g/mL	8 *μ*g/mL

**Table 2:** Size distribution statistical parameters of ZVI materials in nm.

Sample	Mean	CV (%)	D10	D50	D90	D50-D10
AgNPs	10.87	22.94	7.12	10.54	13.72	3.42

**Table 3:** Dynamic light scattering (DLS) and zeta potential of Ag sample.

Sample	Particle size (nm)	Z-potential (mV)
AgNPs	16.46 ± 0.46	−27.3 ± 1.22

**Table 4:** Minimum inhibitory concentrations of Ag nanomaterials.

MIC of silver nanoparticles (mg/ml)			
Sample	*E. coli* (ATCC 25922)	Bacterial strains *S. aureus* (ATCC 29213)	*E. faecalis* (ATCC 29212)

AgNPs	6.68 ± 0.0	13.36 ± 0.0	26.72 ± 0.0
5-Fluorouracil	7.8 ± 0.0	7.8 ± 0.0	15.62 ± 0.0
AgNPs+ 5-FU	1.95 ± 0.0	0.97 ± 0.0	0.97 ± 0.0
*Annona muricata *extract	−a	−a	−a
Amikacin	1 ± 0.0	2 ± 0.0	128 ± 0.0

## Data Availability

All the data used to support the findings of this study (figures and tables) are included within the article.

## Supplementary Materials

Supplementary Materials Schematic illustration representing the synthesis, functionalization, antimicrobial, and cytotoxic effects of silver nanoparticles.
