# Supplementary figures and images for: Cytotoxic and Bactericidal Effect of Silver Nanoparticles Obtained by Green Synthesis Method Using Annona muricata Aqueous Extract and Functionalized with 5-Fluorouracil
Source: Bioinorg Chem Appl. 2018 Oct 15;2018:6506381. doi: 10.1155/2018/6506381 (PMC6205100; doi:10.1155/2018/6506381)

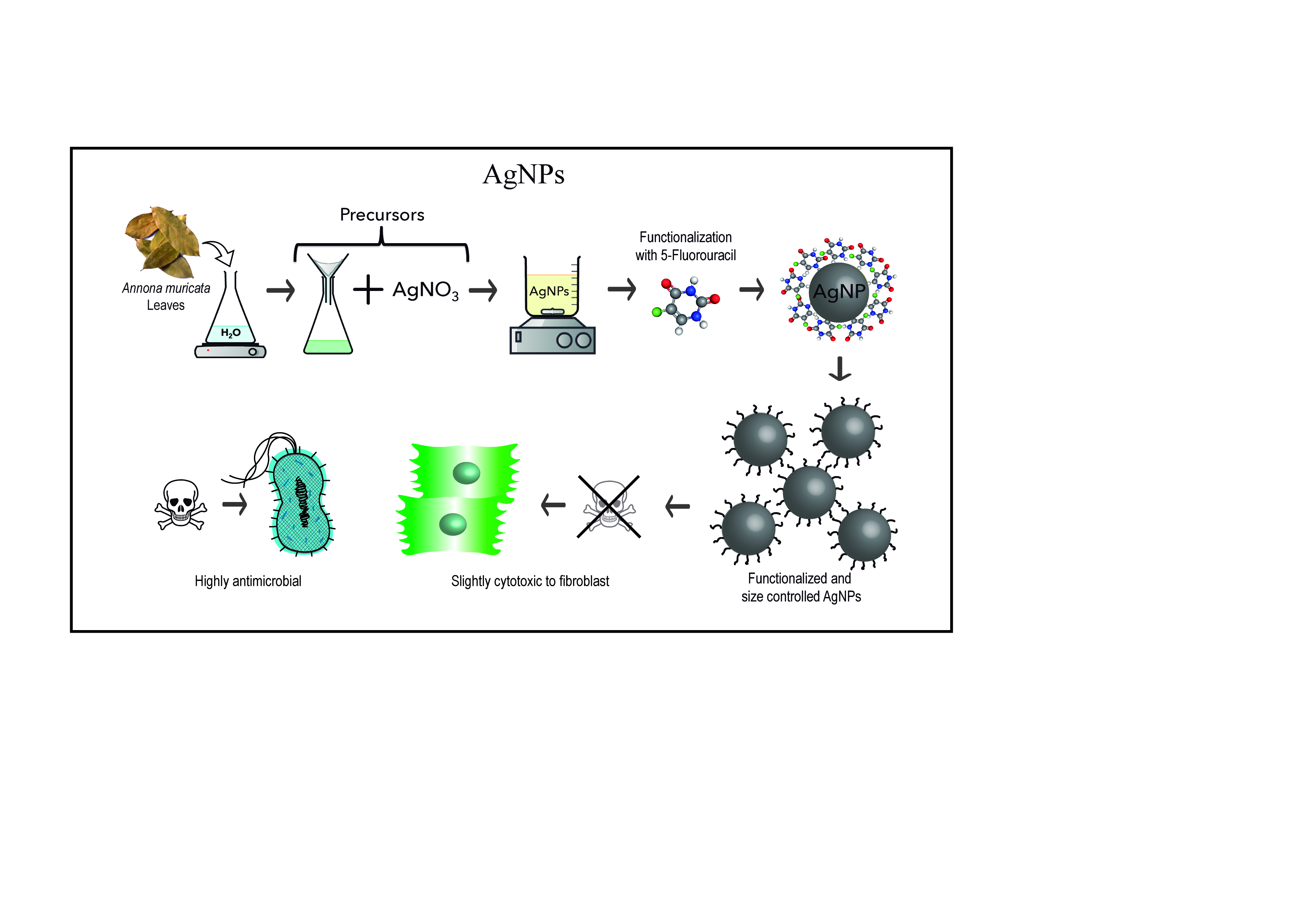

Supplement: Supplementary Materials — Schematic illustration representing the synthesis, functionalization, antimicrobial, and cytotoxic effects of silver nanoparticles. [file 6506381.f1.tif]
